# Adherence to the United States Preventative Services Task Force Female Screening Guidelines: An Internal Medicine Residency Program Experience

**DOI:** 10.7759/cureus.5127

**Published:** 2019-07-12

**Authors:** Chikezie K Alvarez, Julvet Mbianda, Muhammad Nadeem, Shahryar Anwar Ansari, Ahmed Dirweesh, Mohammad Arsalan, Shaikh Fawwad, Vincent A DeBari, Donald Christmas, Sara L Wallach

**Affiliations:** 1 Medicine, St. Francis Medical Center, Trenton, USA; 2 Internal Medicine, St. Francis Medical Center, Trenton, USA; 3 Internal Medicine, Hackensack Meridian School of Medicine at Seton Hall University, Nutley, USA

**Keywords:** mammogram, dxa scan, united states preventive services task force, breast cancer screening, osteoporosis screening

## Abstract

Background

Various guidelines exist for female preventative screening tests and medical resident physician adherence to the United States Preventive Services Task Force (USPSTF) guidelines varies. National screening rates for breast cancer and osteoporosis have improved but they are still below the expected target.

Material and methods

Ambulatory medical clinic records of female patients from the period July 2015 to December 2017 were reviewed for breast cancer and osteoporosis screening. Resident performance and commitment with regards to ordering the aforementioned screening tests according to the USPSTF guidelines were compared to the most recent national screening rates for mammograms and dual-energy X-ray absorptiometry (DXA) scans.

Results

Of the 1327 charts reviewed, 1025 was included in the study. Of the 545 mammograms performed, 93% of them were indicated according to the USPSTF guidelines (P < 0.0001, 95% CI: 125.9-342.0). A total of 480 mammograms were not ordered, of which 6% were indicated and 93.9% were not indicated. Out of a total of 107 DXA scans performed, 88.7% were correctly indicated (P < 0.0001, 95% CI: 37.11-132.9).

Conclusion

Resident physician adherence to the USPSTF screening guidelines for breast cancer and DXA scans were higher than the national and state screening rates. Our well-structured educational project (strong faculty mentorship, resident to patient continuity of care and the reasonable resident-clinic load) resulted in higher screening rates.

## Introduction

In 2016, United States Preventive Services Task Force (USPSTF) issued an update to their 2009 breast cancer screening recommendations. The changes were subtle regarding women aged 50-74 [1]. Their most recent updates to osteoporosis and cervical cancer screening were published in 2011 and 2012, respectively [2,3]. Various societies and organizations have similar but sometimes varying degrees of screening guideline parameters for breast cancer and osteoporosis prevention. Multiple studies have shown varying degrees of physicians’ adherence to cancer screening guidelines issued by USPSTF [4-7]. They also showed that residents tend to have a low performance with prevention screening. This performance improves with experience [5]. As a teaching institution, we performed a chart review to assess our residents’ adherence to preventive testing to look for training improvement opportunities. Our aim was to assess our resident physician performance and adherence to the recommended screening guidelines for breast cancer and osteoporosis according to the USPSTF guidelines. We also compared our screening rates to the national screening rates for breast cancer and osteoporosis. We share our findings to provide an insight into a community-based teaching institutions’ experience and also to shed some light into the challenges that face similar programs.

## Materials and methods

The study was approved by the Institutional Review Board (IRB). This was a cross-sectional study which retrospectively reviewed ambulatory medical clinic female records (n = 1025) from the period July 2015 to December 2017 at an urban teaching healthcare facility in New Jersey, USA and it was approved by the IRB. Charts were reviewed to assess resident physician adherence to the USPSTF screening guidelines for cervical cancer and osteoporosis via screening mammography, and dual-energy X-ray absorptiometry (DXA) scans.

Resident performance and commitment with regards to ordering the aforementioned screening tests when applicable according to the USPSTF guidelines were compared to the most recent national screening rates for mammograms and DXA scans [8-10]. This quality improvement project utilized this time period so that an assessment can be made of the current PGY-1, PGY-2 and PGY-3 internal medicine residents class. We included individuals who completed DXA and mammogram testing, as confirmed by the hospitals’ electronic medical records (EMR). Charts were also reviewed to assess residence adherence to pap testing; however we chose not to include it in our analysis as we could not verify if they were completed by patients. Patients performed pap tests at an external outpatient office and the confirmatory results were not available to us via our EMR. As a result, we could not directly confirm whether pap tests were performed. We sought to assess whether the appropriate screening tests were ordered and performed if indicated, as well as if testing was done even when it was not appropriately indicated. Total of 1,327 charts were reviewed, of which 1,025 charts were selected for inclusion in our study after meeting the predefined set inclusion and exclusion criteria.

Inclusion criteria

Females >21 years of age who presented to our ambulatory clinic from July 2015 to December 2017 and were seen by medical residents. We included females whose mammogram and osteoporosis screening test were documented in our hospitals’ clinic EMR records.

Exclusion criteria

Females <21 years of age, inpatient population, all males, women with a family history of breast cancer, personal history of breast cancer, known BRCA1/BRCA2 mutations or a history of chest radiation between ages 10 and 30 years (for breast cancer screening). Corticosteroid use >3 months, family history of osteoporosis, previous fracture, women <65 years with a FRAX score >9.3%, parental history of hip fracture, greater than two drinks alcohol/day, rheumatoid arthritis, current BMI < 20 (for osteoporosis screening). Charts of patients who were not seen by residents were excluded. Charts of patients who did not have documented proof of completed mammograms and DXA scans were excluded.

Results were computed into three separate 2 x 2 contingency tables for screening mammography, and DXA scans. Descriptive statistics with cross-tabulations were performed. The Fisher exact test was used to examine the association between variables. Odds ratio was used as a measure of effect size. Comparisons which yielded P-values less than 0.05 on a two-sided basis were considered statistically significant.

## Results

Of the 545 mammograms ordered and completed, 93% (507 of 545) of them were indicated according to the USPSTF guidelines and 6.97% (38 of 545) were not indicated. A total of 480 mammograms were not ordered, of which 6% (29 of 480) were indicated and 93.9% (451 of 480) were not indicated (P < 0.0001) (Table 1).

**Table 1:** Mammography Results

	Mammogram Performed	Mammogram Not Performed	Total
Mammogram Indicated	507	29	536
Mammogram Not Indicated	38	451	489
Total	545	480	1025
P < 0.0001 (95% CI: 125.9-342.0). Odds ratio: 207.5			

Out of a total of 107 DXA scans ordered and completed, 88.7% (95 of 107) were correctly indicated and 11.2% (12 of 107) were not (P < 0.0001). A total of 918 DXA scans were not ordered, of which 10.1% (93 out of 918) DXA scans were indicated according to the USPSTF guidelines and 89.8% (825 out of 918) DXA scans not indicated (P < 0.0001) (Table 2).

**Table 2:** DXA Results DXA: Dual-energy X-ray absorptiometry; CI: Confidence interval.

	DXA Performed	DXA Not Performed	Total
DXA Indicated	95	93	188
DXA Scan not Indicated	12	825	837
Total	107	918	1025
P < 0.0001 (95% CI: 37.11–132.9). Odds ratio: 70.23			

## Discussion

The aim of this study was to assess our institutions’ resident physician adherence to the USPSTF screening guidelines with regards to mammograms and DXA scans. The demographic population of our medical clinic consists of 40% Hispanic, 30% Black, and 30% “Others”. We also serve and treat a significant indigent population with our ambulatory medical clinic consisting of 50% uninsured individuals on charity care, 40% on managed Medicaid and 10% on Medicare.

The burden of breast cancer is still significant, as there are approximately 125 new cases of breast cancer and about 22 deaths per 100,000 U.S. women each year [1]. The USPSTF recommends biennial screening mammography for women aged 50 to 74 years [1]. Although other societies recommend screening from age 40, studies have shown that the cumulative rates of false-positive mammography results are higher with annual rather than biennial screening (mammography, 61% vs. 42%), for women with heterogeneously or extremely dense breasts, and those aged 40 to 49 years [11]. Our study revealed a significant adherence to the USPSTF screening guidelines for breast cancer as 93% of mammograms ordered by resident physicians were performed and done according to the guidelines.

According to the Centers for Disease Control and Prevention (CDC), national screening rates for mammograms were 67.2% with 17.3 million tests performed in 2016 [12]. Resident screening rates were greater than the national average according to the National Cancer Institute which showed that in 2015, 71.6% of women aged 50-74 years had a mammogram within the past two years [8]. At the state level, our screening rates were higher than that of New Jersey which was 78.2% which in itself is below the “Healthy People 2020” screening target of 81.1% [13,14].

One possible reason for the high resident adherence to the screening guidelines is that at each female clinic encounter, residents are required to document patients’ most recent screening mammogram for females aged 50-74 years which helps to ensure that mammography screening testing is addressed at each visit. In addition, we were able to access the results for prior mammograms (if applicable) via our EMR to help improve the validity. Although only 6.97% of the mammograms performed were done so inappropriately, this may be due to residents not adhering to the screening intervals, according to the USPSTF guidelines. Screening guidelines from other societies may have been used inadvertently at times, such as the American Cancer Society, which recommends annual mammograms from age 45 [15].

The USPSTF recommends screening for osteoporosis in women aged 65 years or older [3,16]. DXA scan is the gold standard for diagnosing osteoporosis and also as a guide for deciding which patients to treat. Osteoporosis imparts a significant cost burden, with direct medical costs in the United States estimated at $12-20.3 billion dollars [17,18]. National DXA screening rates according to the CDC were 24.5% with a sample size of 5.8 million scans [19,20]. One study which looked at a cohort of 1,638,454 female patients, screening rates were very low; 26.5%, and 12.8% among women aged 65-79, and 80+ years, respectively [10]. Rates in a much smaller retrospective cohort study which sampled 50,995 women found that the incidence of DXA screening was 57.8% for women aged 65-74, and 42.7% for women aged ≥75 years [21].

Results from our study show that 107 DXA scans were performed with 88.7% (95 of 107) of them being ordered appropriately according to the USPSTF recommendations. Although this is significantly higher than the screening rates from the two previously mentioned studies, 11.2% of the DXA scans ordered were done inappropriately. In addition, 10.1% of DXA scans, which were not ordered, were done inappropriately as they should have been ordered, according to the guidelines. This is an area for improvement with regards to resident knowledge and adherence to the USPSTF guidelines for osteoporosis screening as fractures cause premature mortality, loss of independence and function and a reduced quality of life. One additional potential reason for the increased lack of our resident adherence to osteoporosis screening compared to mammograms and pap tests is the lack of a clearly defined interval for repeating DXA scans. According to the USPSTF, a lack of evidence exists about optimal intervals for repeated screening and whether repeated screening is necessary in a woman with normal bone mineral density (BMD) [22].

There are several limitations of our study such as the small sample size of our cohort of patients and the single center design. In addition, screening pap tests were done at an external outpatient office and we could not retrieve the results via our EMR. As a result, we did not include these results in our analysis.

Even with these limitations, our experience can provide a template to increase screening rates in the general population. Our recommendation as a single center based on our experience is for strong resident mentorship and close faculty monitoring. In addition, limiting the patient load for each resident on their respective clinic sessions allows residents to dedicate and spend more time reviewing the heath care maintenance aspect of each patient encounter. Also, with our significant Hispanic population an in-person Spanish translator was added in December 2016, who is medically certified for translation services. This has allowed for better and more effective physician to patient communication regarding the importance of screening for mammograms, pap tests and DXA scans.

Where possible, a certified inpatient language translator can be a very beneficial addition, which can minimize some of the inherent limitations of phone translation. This has improved patient compliance and allowed for an improved patient to physician interaction. Lastly, one of the most important factors which improves screening rates is the continuity of care provided in the program by the residents and the attending physicians. Our clinic was divided into various teams, each with their respective patients which allows residents and attending physicians to closely monitor all of the respective screening tests for their assigned patients.

## Conclusions

Resident physician adherence to the United States Preventive Services Task Force screening guidelines for breast cancer and osteoporosis was high compared to the national and state screening rates. Implementation of an educational protocol consisting of strong faculty mentorship, continuity of care between residents, attending physicians and patients, the addition of an in-person medically certified Spanish language translator, as well as managing resident patient load so as to allow for a thorough patient encounter, can help to overcome some of the boundaries that individuals from low socioeconomic status may face.
